# Photosynthetic Pigments in Diatoms

**DOI:** 10.3390/md13095847

**Published:** 2015-09-16

**Authors:** Paulina Kuczynska, Malgorzata Jemiola-Rzeminska, Kazimierz Strzalka

**Affiliations:** 1 Faculty of Biochemistry, Biophysics and Biotechnology, Department of Plant Physiology and Biochemistry, Jagiellonian University, Gronostajowa 7, Krakow 30-387, Poland; E-Mails: kuczynska.paul@gmail.com (P.K.); malgorzata.jemiola@gmail.com (M.J.-R.); 2 Małopolska Centre of Biotechnology, Gronostajowa 7A, Krakow 30-387, Poland

**Keywords:** bioactive compounds, biosynthesis pathway, diatoms, photoprotection, photosynthesis, pigments

## Abstract

Photosynthetic pigments are bioactive compounds of great importance for the food, cosmetic, and pharmaceutical industries. They are not only responsible for capturing solar energy to carry out photosynthesis, but also play a role in photoprotective processes and display antioxidant activity, all of which contribute to effective biomass and oxygen production. Diatoms are organisms of a distinct pigment composition, substantially different from that present in plants. Apart from light-harvesting pigments such as chlorophyll a, chlorophyll c, and fucoxanthin, there is a group of photoprotective carotenoids which includes β-carotene and the xanthophylls, diatoxanthin, diadinoxanthin, violaxanthin, antheraxanthin, and zeaxanthin, which are engaged in the xanthophyll cycle. Additionally, some intermediate products of biosynthetic pathways have been identified in diatoms as well as unusual pigments, e.g., marennine. Marine algae have become widely recognized as a source of unique bioactive compounds for potential industrial, pharmaceutical, and medical applications. In this review, we summarize current knowledge on diatom photosynthetic pigments complemented by some new insights regarding their physico-chemical properties, biological role, and biosynthetic pathways, as well as the regulation of pigment level in the cell, methods of purification, and significance in industries.

## 1. Introduction

Diatoms are becoming more and more prominent microalgae. More advanced knowledge about them has also enhanced their importance and usefulness in commercial and industrial applications such as biofuels, pharmaceuticals, health foods, biomolecules, materials relevant to nanotechnology, and as bioremediators of contaminated water [1]. The singularity of these organisms is physiological in nature, involving, for example, novel metabolic pathways and compounds, but is also due to their importance in the evolutionary history of eukaryotes as well as their ecological success. They are mainly associated with the silicon metabolism engaged in the biogeochemical cycling of Si in the sea. The siliceous structures in their cell wall create the unique morphotypes that are used as taxonomic keys [2]. Another interesting process is the ornithine-urea cycle which is absent in green algae and plants and is essential for diatom growth and their contribution to marine productivity [3]. Furthermore, these organisms are rich in bioactive compounds capable of antiviral activity, including naviculan [4] and neuroexcitatory amino acid derivative domoic acid [5], not to mention the cytotoxic and blood platelet inhibitory activity caused by adenosine [6]. There is a high diversity of beneficial diatom cell components in lipids and pigments, whose amount in the cell may be partially regulated by certain abiotic stresses or genetic modifications of metabolic pathways. For example, the overexpression of endogenous Δ5 desaturase leads to an accumulation of eicosapentaenoic acid, a fatty acid known to have a variety of health benefits including anti-inflammatory effects and better neuronal functioning [7]. On the other hand, enhanced biosynthesis of strong antioxidant fucoxanthin was obtained through low light and nitrogen treatment [8]. Although not all the compounds in diatoms are known, due to the importance of these organisms, there is continual research to find, identify, and examine their properties. In the marine diatom *Haslea ostrearia*, a water soluble blue pigment named marennine was identified and further experiments have shown its allelopathic, antioxidant, antibacterial, antiviral, and growth-inhibiting properties [9]. Another diatom, *Haslea karadagensis*, also has a blue pigment. Although it has quite different absorption maxima from those of marennine, its similar bioactivity has come to be called marennine-like [10]. While these unusual pigments occur only in selected species, there are some which are included in the more general group of photosynthetic pigments and are common among diatoms. These also have great benefits in medicine, pharmacy, cosmetics, food, and supplements. However, the pigment profile of diatoms is quite different than that found in plants and some algae. Chlorophyll a (Chl *a*) and chlorophyll c (Chl *c*) together with fucoxanthin (Fx) are components of fucoxanthin-chlorophyll protein (FCP) complexes which replace plants light harvesting complexes (LHC) in performing the light-harvesting function [11]. Furthermore, the three carotenoids—β-carotene (β-car), diadinoxanthin (Ddx), and diatoxanthin (Dtx)—are known to play an important role in photoprotection and, additionally, violaxanthin (Vx), antheraxanthin (Ax), and zeaxanthin (Zx) may be engaged in this process. This article is focused on the above-described photosynthetic pigments which are essential for diatom life and which are commonly used in various industries. Despite the fact that studies in this field have been carried out for many years, a lot of aspects that require further analysis still remain. What follows below represents the current knowledge of their physical and chemical properties, their biosynthetic pathways, their regulation of pigment level, as well as their localization in the cell, their role in photosynthesis and photoprotection, methods for their identification and purification, and their significance in industries.

## 2. Physical and Chemical Properties of Photosynthetic Pigments of Diatoms

Diatoms contain two types of pigments involved in light harvesting and photoprotection: chlorophylls and carotenoids. Chlorophylls trap light energy—blue and red portions of the electromagnetic spectrum, in particular, which are used in photosynthesis. Generally, chlorophylls can be defined as a magnesium coordination complex of cyclic tetrapyrroles containing a fifth isocyclic ring, referred to as porphyrin, with a long-chain isoprenoid alcohol ester group. Being the highly conservative structural motif of Chls, however, the phytyl chain is not present in the majority of Chl *c* pigments found in diatoms. The second group of pigments, carotenoids, are engaged mainly in photoprotection; however, Fx also participates in light harvesting. They are comprised of carotenes (hydrocarbons) and their oxygenated derivatives, xanthophylls. Extensive data compiled for 47 of the most important chlorophylls and carotenoids found in marine algae were published by Jeffrey and Vesk [12].

### 2.1. Chlorophylls

**Figure 1:**
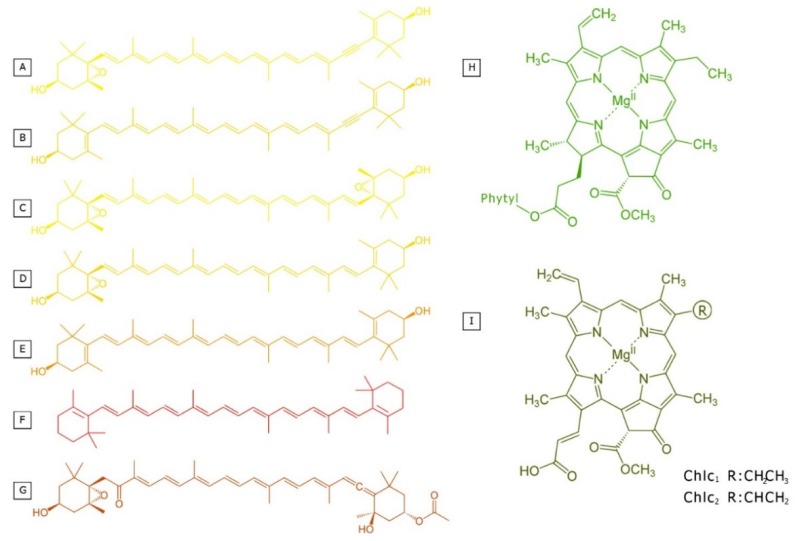
Structural formula of photosynthetic pigments in diatoms including *all-trans* carotenoids: (**A**) diadinoxanthin; (**B**) diatoxanthin; (**C**) violaxanthin; (**D**) antheraxanthin; (**E**) zeaxanthin; (**F**) β-carotene; (**G**) fucoxanthin; and chlorophylls: (**H**) chlorophyll *a*; (**I**) chlorophyll *c*.

Several kinds of chlorophylls are found in photosynthetic organisms; however, only two forms occur in diatoms: Chl *a* and, identified in various algae, Chl *c*. The predominant Chl *a* plays a central role in the photochemical energy conversion of the majority of photosynthesizing organisms, while Chl *c* participates effectively in photosynthesis as an accessory pigment, similar in its functional activity to the Chl *b* of higher plants. From among different forms of Chls *c* which were described in diatoms, the most abundant are Chl *c*_1_ and *c*_2_ (Figure 1). The distinct structure of a Chl *c* brings changes in the absorption spectrum to produce a strong Soret (blue) absorption band in comparison with a weak band in the red region. The ratios of band I (at ~630 nm) to band II (at ~580 nm) are >1 for Chl *c*_1_-like chromophores, ~1 for Chl *c*_2_-like chromophores, and <1 for Chl *c*_3_-like chromophores [13].

Among 51 species (71 isolates) of tropical and sub-tropical diatoms from 13 out of 22 families examined by Stauber and co-workers [14], Chl *c*_2_ was present in all the diatoms tested and occurred together with Chl *c*_1_ in 88% of them. Where Chl *c*_1_ was absent or occurred in trace amounts only, it was usually replaced by a Chl *c_3_*, identified by Fookes and Jeffrey as (7-methoxycarbonyl)-Chl *c*_2_ [15]. The presence of a methoxycarbonyl group (–COOCH_3_) at C-7 (ring B) explains the difference in molecular weight (653 *m*/*z*) compared to Chl *c_2_* (609 *m*/*z*), as well as the significant decrease in absorption band I (Qy) intensity compared to the Chl *c*_1_ and *c*_2_ values [16]. Exceptions are *Nitzschia closterium* (CS-114), with merely Chl *c*_2_, and *Nitzschia bilobata* (CS-47), which contains all three Chls (*c*_1_, *c*_2_, and *c*_3_) in approximately equal amounts. Five species that have Chls *c*_1_ and *c*_2_ also contain Chl *c*_3_ in trace quantities. Additionally, diatoms may possess the Chl *c*_2_-P.gyrans-type found, for example, in *Pseudo-nitzschia multiseries* [16]. Finally, traces of (DV)-PChlide with a propionic acid side chain at C-17 instead of acrylic acid are commonly present in diatoms. An overview of the Chl *c* distribution in microalgae classes is given by Jeffrey and Vesk [12].

In the aquatic environment, pigments may undergo degradation in response to chemical, photochemical, and biological processes. Studies of marine organic matter from the central equatorial Pacific were able to weigh the overall reactivity of various biochemical classes and found that pigments, especially chlorophylls, were the most labile compounds [17]. However, possessing nitrogen, chlorophylls are more prone to being salvaged during senescence and biological breakdown than carotenoids. Although the photooxidation of chlorophyll was studied almost exclusively in terms of the porphyrin moiety of the molecule, its unsaturated chain is also susceptible to a reaction with singlet oxygen or the hydroxy and peroxy radicals which are generated during Chl photodegradation. It has been demonstrated by Rontani and co-workers [18] that several free and esterified oxidized isoprenoid compounds are produced during the photodegradation of Chl *a* in seawater. Moreover, they suggested that in the dead cells of *Phaeodactylum tricornutum*, the Chl phytyl chain will be reduced to up to one half of its initial concentration after prolonged exposure to high light.

### 2.2. Carotenoids

More than 700 types of carotenoids were identified in nature [19]. They are commonly synthesized by plants, algae, and some micro-organisms. Seven kinds of carotenoids were found in diatoms with β-car as an example of carotenes, as well as Fx, Dtx, Ddx, Zx, Ax, and Vx, which represent xanthophylls (Figure 1). Although derivatives of all mentioned pigments, including isomers and products of degradation, may occur in the cell, the all-*trans*-isomers are the most abundant and functionally active forms. The presence of a conjugated polyene chain in carotenoids may be the cause of carotenoid instability, which is related to their susceptibility to oxidation, E/Z isomerization by heat, light, and chemicals. As a result of the c*is*-isomerization of a chromophore’s double bond there is a slight loss of color, as well as a small hypsochromic shift and a hypochromic effect, accompanied by the appearance of a *cis* peak about 142 nm below the longest wavelength absorption maximum of the *trans*-carotenoid when measured in hexane [16]. Moreover, the geometrical structures of *cis* and *trans* carotenoids, which are differentially oriented in the thylakoid membrane, have an impact on membrane physical properties. Intercalation of carotenoids changes permeability for the oxygen and other small molecules, which is associated with their protective activity [20,21]. The activation energy for allenic (R/S) isomerization in carotenoids is higher than that associated with geometrical (E/Z) isomerization. Consequently, E/Z stereomutation of Fx can occur in sunlight in the absence of a catalyst, whereas allenic isomerization was shown to occur only to a very low extent. Recently, data has been published on the iodine or diphenyl diselenide-mediated photoisomerization of Fx, which, in view of the increased reaction rate with UVA radiation, support the radical mechanism of R/S isomerization [22]. Additionally, some carotenoids are highly unstable in the presence of acid. Under weak acidic conditions, 5,6-epoxide is readily rearranged into furanoid 5,8-epoxide. On the other hand, the lability of Fx towards alkali has been established, which precludes the use of saponification in the isolation procedure of carotenoid mixture containing Fx. First, chromophoric changes upon treatment of this allenic carotenoid with a weak base (K_2_CO_3_) were reported and, then, two products were identified: (i) isofucoxathinol as the kinetically controlled product and (ii) fucoxanthinol hemiketal, with a shorter chromophore, as the thermodynamically controlled product [23]. Unlike chlorophylls, carotenoids are often broken down into a colorless compound by the destruction of the long chain of alternating double bonds and cannot be detected by regular pigment analysis [24].

Carotenoids exhibit intense absorption between 400 and 500 nm. The conjugation length and type of the functional groups that are attached to the ionone rings terminating the polyene chain largely determine the absorption properties of the carotenoids [17,25]. The absorption spectra of carotenoids are markedly solvent-dependent, which should be considered when analyzing pigment extracts by high performance liquid chromatography with a photodiode array detector (HPLC-DAD), because in most cases spectra are taken in mixed solvents. The λ_max_ values of carotenoids in hexane or petroleum ether are practically the same as in diethyl ether, methanol, ethanol, and acetonitrile, and are higher by 2–6 nm in acetone, 10–20 nm in chloroform, 10–20 nm in dichloromethane, and 18–24 nm in toluene; see [16] for ultraviolet and visible absorption data for common carotenoids. To give an idea of the spectral fine structure, the values of %III/II are also given along with the λ_max_ values.

The main light-harvesting carotenoid in diatoms is Fx, which is also abundant in brown algae. Unusually, small amounts of a 19′-butanoyloxyfucoxanthin-like pigment, in addition to Fx, were found in one diatom species, *Thalassiothrix heteromorpha*, as reported in [26]. Fx has an allenic bond, a conjugated carbonyl, a 5,6-monoepoxide, and acetyl groups that contribute to the unique structure and spectral properties of the molecule (Figure 1). In contrast to other carotenoids, its broad absorption band (between 460 and 570 nm) covers much of the gap left by chlorophyll in the green region. Diatoms also possess the β-car of carotenes as well as two xanthophylls, Ddx and Dtx, which are also asymmetric molecules, containing an acetylenic group at one of the ionone rings. Moreover, three other xanthophylls, characteristic of higher plants, Vx, Ax, and Zx, may also occur (Figure 1) [24,27]. However, these carotenoids accumulate only under specific conditions, e.g., during long-time illumination with strong light (see Section 8).

## 3. Biosynthesis Pathways

The biosynthetic pathways of both chlorophylls and carotenoids in plants have been extensively studied and complete information in this field is available. This opens up many opportunities for studies on genetically modified organisms and for *in vitro* approaches using recombinant proteins. In diatoms, some pathway points remain unclear and the production of cell lines with an enhanced amount of a selected photosynthetic pigment is currently difficult. Most of the genes which encode enzymes in these steps of the pathway were sought out by genome alignment, but they have not been identified. The whole genomes of two diatom species, *Phaeodactylum tricornutum* [28] and *Thalassiosira pseudonana* [29], were sequenced, but few analogues of the genes which occur in plants or algae were found in these diatoms.

**Figure 2:**
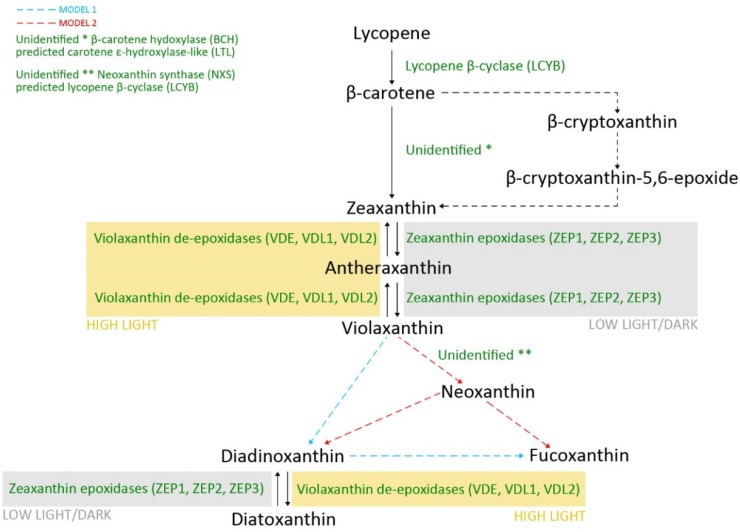
Biosynthetic pathway of photosynthetic carotenoids in the diatom *Phaeodactylum tricornutum* from lycopene to fucoxanthin and diatoxanthin.

Two pathways, methylerythritol phosphate (MEP) and mevalonate (MEV), of the early steps of carotenoid biosynthesis have been described. Their occurrence is not clear but a few studies show that it depends on the taxon or the growth rate. However, the products of both are dimethylallyl diphosphate (DMAPP) and its isomer, isopentenyl pyrophosphate (IPP) [14,16,30]. The next steps on the pathway to lycopene synthesis are the conversion of DMAPP to geranylgeranyl pyrophosphate (GGPP), which is catalyzed by GGPP synthase, then to phytoene by phytoene synthase (PSY), afterwards to ζ-carotene, which is catalyzed by phytoene desaturase (PDS), and, finally, the product of ζ-carotene desaturase (ZDS) is lycopene [30]. The biosynthesis pathway from lycopene to xanthophylls is presented in Figure 2. Firstly, lycopene as a long and straight molecule is cyclized by lycopene β-cyclase (LCYB) to β-car, having two β-ionone rings at both ends of the yield. In the next step, xanthophyll is first formed, and this reaction requires hydroxylation. However, a gene encoding β-carotene hydroxylase (BCH) was not found in the diatom genome and another one that is similar to LUT1 has been proposed as a putative enzyme to make the formation of Zx from β-car possible [31]. Two further light-dependent and reversible reactions lead to Vx formation via the intermediate product Ax. Both are catalyzed by Vx de-epoxidases (VDEs) in high light conditions, but reverse reactions are catalyzed by Zx epoxidases (ZEPs) in low light or in the dark [32]. In *T. pseudonana*, two VDEs and two ZEPs were identified [33], while in *P. tricornutum*, three isoforms of ZEPs (ZEP1, ZEP2, ZEP3) and VDEs (VDE, VDL1, VDL2) were found [31]. Alternatively, Vx might be formed from Zx through β-cryptoxanthin (Cx) and β-cryptoxanthin-epoxide (CxE) [27]. Further steps which lead to the formation of Fx, Ddx, and Dtx are still unclear because of missing data about the enzymes engaged in the process. However, to date, two models of possible conversions from Vx to Fx have been presented. The first model was described by Lohr and Wilhelm [27], who proposed Vx as a precursor of Ddx, Dtx, and Fx, with the reaction from Vx to Dtx as well as to Fx proceeding via Ddx. This hypothesis was confirmed experimentally using norflurazon, which inhibits the *de novo* synthesis of carotenoids and which was used after the accumulation of Vx. In low light, an increase in the Fx level was detected. The other model is based on speculation about the chemical properties of these xanthophylls and neoxanthin (Nx) was regarded as a precursor of both Ddx and Fx [34]. Nx is the most likely candidate for enabling the formation of the acetylenic bond in Ddx or the allenic double bond in Fx. However, the formation of Fx requires two modification steps: the ketolation of Nx and the acetylation of an intermediate, probably fucoxanthinol. To support one of these hypotheses, the identification of the enzymes is necessary. The most powerful approach in this field is to look for genes encoding the proteins of interest on databases. Unfortunately, the gene encoding Nx synthase (NXS), which catalyzes the conversion of Vx to Nx in *Arabidopsis thaliana*, was not found in brown seaweeds. Moreover, Nx has not been detected either. However, LCYB shares a 64% amino acid identity with NXS and is proposed to be engaged in Nx production, although no LCYB-like NXS in brown seaweeds was identified [35]. It is important to reveal the whole xanthophyll biosynthetic pathway because of the many opportunities to further studies and also to prepare transgenic organisms with an increased xanthophyll level.

The chlorophyll biosynthetic pathway has been extensively studied in higher plants and also in some groups of algae, but in the case of diatoms, it has been poorly investigated. Nevertheless, the main aspects are similar in all photosynthetic organisms. Three general steps are needed for Chl synthesis: aminolevulinic acid formation, its transformation into Mg-porphyrins, and protochlorophyllide conversion to Chl [36]. The initial steps rely on tetrapyrrole cyclization, the insertion of Mg leading to diviny-PChlide *a* formation, and its reduction to PChlide *a*. Afterwards, light-independent PChlide oxidoreductase (DPOR) and a light-dependent enzyme (LPOR) catalyze PChlide hydrogenation to Chlide and further steps lead to Chl *a* formation. In four diatom species, more than one isoform of LPOR was found [37]. The final step is the introduction of phytol residue, which is associated with the MEP pathway also used in carotenoid formation [38]. In higher plants, the chlorophyll cycle relies on conversion between Chl *a* and Chl *b*, allowing the adjustment of their ratio to light conditions [39]; however, in diatoms, instead of Chl *b*, Chl *c* (Chl *c_1_*, Chl *c_2_*, and rarely Chl *c_3_*) was identified [13,14] (see also Section 2). The molecular structure of Chl *c* may suggest that PChlide is its precursor in the biosynthetic pathway where oxidation and dehydration are required, but no enzyme carrying out these steps has been described [40]. In general, data about Chl biosynthesis and the enzymes which catalyze each step are still poorly understood.

## 4. Regulation of Pigment Level in the Cell

Changes in photosynthetic pigment levels are usually a fast response to environmental conditions because they are engaged in basic processes such as photosynthesis and photoprotection, which are essential for cell life (Table 1). Diatom cell physiology displays some significant differences that are observed during growth, including an exponential, stationary, and declining phase. In the exponential phase of growth, the metabolism is the highest, especially that of amino acids, whereas in the declining phase, catabolism is predominant and is related to an increase in metabolites like terpens and putrescine [41]. Experiments which concern cell responses to stress factors and pigment measurements should be performed during the exponential phase of growth.

Pigment level is mostly regulated by light conditions, resulting in fast conversions of them, usually without any changes in gene expression, although long-term acclimation causes changes in the transcript level of the genes which encode proteins in biosynthetic pathways. The effect of white and blue-green light on pigment content during the exponential and stationary growth phases of three diatom species was studied [42] and it was reported that Chl *a* content decreased by nearly 50% in the stationary phase and carotenoid content increased in blue-green light. In high light, the amount of Chl *a*, Fx, and β-car decreased, but with unrelated variations in Chl *c*, and it was also reported that the Chl *c* content in FCP complexes might be regulated by light, but not that of Chl *a* and Fx [43]. Under low light, FCP trimers contain mainly Lhcf5 proteins with a high Fx:Chl *c* ratio, whereas trimers in high light contain mainly Lhcf4 proteins with a low Fx:Chl *c* ratio. A different variation of Chl *a*, β-car, and Fx is correlated with a decreasing number of PSII units in high light. This photoacclimation strategy described in species growing in variable light conditions enables the efficient regulation of photosystem structures to the amount of absorbed energy [43,44]. The spectral composition of light plays a crucial role in growth rate, photoprotective mechanisms, and photosynthetic efficiency, and thereby in pigment content. In blue light-acclimated *P. tricornutum* cells, the pool of xanthophyll cycle pigments was higher than those growing in red light, but in neither case was Dtx detected [45]. Studies on the long-term dark incubation of diatoms in the sediment from a tidal mud flat showed that after one year of microscope observations, fluorescence was detected. Pigment analysis including Fx, Chl *c*, Chl *a*, Dtx, and Ddx showed a rapid decrease in the first weeks and, then, slower changes, and although Ddx was not detected after two months, Dtx remained steady over time [46]. It is not only light conditions that have an impact on pigment levels, but also nutrient limitation along with the heavy metals which are more and more abundant in the environment [47]. Iron limitation results in a wide range of changes in diatoms, including in gene expression in factors which regulate pigment content. As many as 20 genes of iron-responsive regulation were described in *P. tricornutum* [48]. It has been reported that under iron limitation, there was an increase in the transcript level of 16 genes involved in Chl biosynthetic pathways, although the Chl content decreased [49]. With an increased cadmium concentration, a rapid decrease in Chl *a* as well as carotenoids was observed in epiphytic diatoms of *Myriophyllum triphyllum* [50]. Although the pigment composition is similar in each diatom species, the ratio between them is different and highly variable. Unfortunately, a comparison of pigment content in different diatom species growing under optimal or stress conditions is very complicated because of the variety of ways in which it is calculated. This is very often done in relation to Chl *a* content, but the absolute content in dry or wet weight is also measured, or the percentage of each pigment is measured.

**Table 1:** Changes in pigment content (Chl *a*: chlorophyll *a*; Chl *c*: chlorophyll *c*; β-car: β-carotene; Fx: fucoxanthin; Ddx: diadinoxanthin; Dtx: diatoxanthin; Vx: violaxanthin; Ax: antheraxanthin; Zx: zeaxanthin) in diatoms in response to selected stress conditions. The down arrow represents a decrease of pigment content, the up arrow is the opposite, and *const* means no changes.

Conditions	Species	Changes in Pigment Content								
		Chl *a*	Chl *c*	β-Car	Fx	Ddx	Dtx	Vx	Ax	Zx
HL (140 μmol photons m^−2^·s^−1^) in comparison to LL (40 μmol photons m^−2^·s^−1^), 16 h light/8 h dark photoperiod [51]	*Cyclotella meneghiniana*	N/A	*const*	*const*	*const*	↑	↑	N/A	N/A	N/A
Iron-replete medium (12 μM) compared to iron-reduced medium (1 μM) in HL (140 μmol photons m^−2^·s^−1^) [52]	*Cyclotella meneghiniana*	↑	N/A	N/A	N/A	↓		N/A	N/A	N/A
Iron-replete medium (12 μM) compared to iron-reduced medium (1 μM) in LL (40 μmol photons m^−2^·s^−1^) [52]	*Cyclotella meneghiniana*	↑	N/A	N/A	N/A	*const*		N/A	N/A	N/A
HL (300 μmol photons m^−2^·s^−1^) compared to LL (50 μmol photons m^−2^·s^−1^), 14 h light/10 h dark photoperiod [53]	*Phaeodactylum tricornutum*	N/A	↓	↑	↓	↑	↑	N/A	N/A	N/A
B-HL (450 PFD) compared to BR-HL (450 PFD in R:B ratio 0.25) [43]	*Pseudonitzschia multistriata*	↓	↑	*const*	↓	↓	↓	N/A	N/A	N/A
B-LL (250 PFD) compared to BR-LL (250 PFD in R:B ratio 0.25) [43]	*Pseudonitzschia multistriata*	↓	↑	*const*	↓	↓	↓	N/A	N/A	N/A
B-LL (24 (10 absorbed) μmol photons m^−2^·s^−1^) compared to W-LL (40 (10 absorbed) μmol photons m^−2^·s^−1^) [54]	*Phaeodactylum tricornutum*	↑	*const*	*const*	*const*	*const*	N/A	↓	N/A	N/A
R-LL (41 (10 absorbed) μmol photons m^−2^·s^−1^) compared to W-LL (40 (10 absorbed) μmol photons m^−2^ s^−1^) [54]	*Phaeodactylum tricornutum*	↑	↓	↑	↓	↓	N/A	↓	N/A	N/A
HL (1250 μmol photons m^−2^·s^−1^) in comparison to LL (40 μmol photons m^−2^·s^−1^), 12 h light/12 h dark photoperiod [55]	*Phaeodactylum tricornutum*	↓	*const*	*const*	*const*	↓	↑	N/A	N/A	N/A
6 days acclimated to shift from BL (24 (10 absorbed) μmol photons m^−2^·s^−1^) to RL (40 (10 absorbed) μmol photons m^−2^·s^−1^) [45]	*Phaeodactylum tricornutum*	↑	N/A	N/A	N/A	↓	N/A	N/A	N/A	N/A
6 days acclimated to shift from RL (40 (10 absorbed) μmol photons m^−2^·s^−1^) to BL (24 (10 absorbed) μmol photons m^−2^·s^−1^) [45]	*Phaeodactylum tricornutum*	*const*	N/A	N/A	N/A	↑	N/A	N/A	N/A	N/A
14 days dark storage culture [56]	*Thalassiosira weissflogii*	↓	↓	↓	↓	↓		N/A	N/A	N/A
HL (700 μmol photons m^−2^·s^−1^) in comparison to LL (40 μmol photons m^−2^·s^−1^), 16 h light/8 h dark photoperiod [24]	*Cyclotella meneghiniana*	N/A	N/A	N/A	N/A	↓	↑	*const*	↑	↑
high nitrogen culture (18 mM) compared to low nitrogen culture (6 mM) in LL (100 μmol photons m^−2^·s^−1^) [8]	*Odontella aurita*	N/A	N/A	N/A	↑	N/A	N/A	N/A	N/A	N/A

B-HL: high blue light; B-LL: low blue light; BR-HL: high blue/red light; BR-LL: low blue/red light; HL: high light; LL: low light; PFD: photon flux density; R-LL: low red light; W-LL: low white light; N/A: not available.

## 5. Localization in the Cell

**Figure 3:**
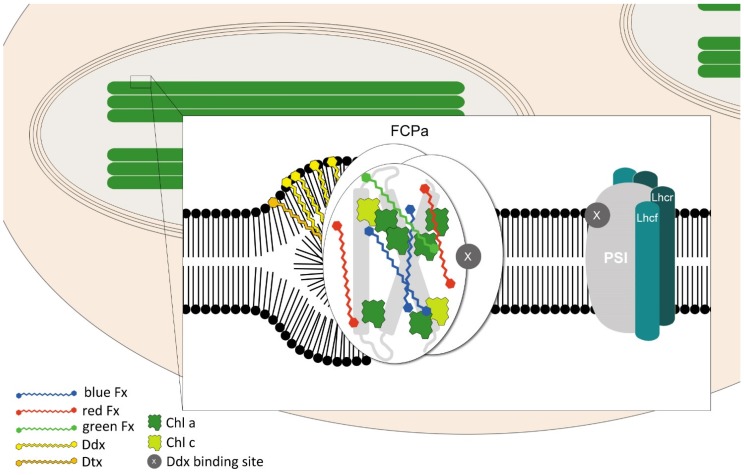
Simplified model of diatom thylakoid membrane showing the localization of photosynthetic pigments within FCP, PS I, and those localized within an monogalactosyldiacylglycerol (MGDG) shield surrounding the FCP. See the text for more information. Based on Gundermann and Büchel [60].

Diatom cells contain either a few small chloroplasts or one large chloroplast [57]. In diatoms, the thylakoid membranes, where pigments responsible for the absorption of light for photosynthesis are located, are not differentiated into granal and stromal lamellae, *i.e.*, granal stacking is absent [58]. Instead, the diatom thylakoid membranes are arranged into groups of three loosely stacked lamellae which span through the whole length of the chloroplast (Figure 3) [59]. Consequently, no lateral heterogeneity in the distribution of photosystems (PS) I and PS II has been detected so far. The organization of the LHC proteins, recently reviewed in detail by Gundermann and Büchel [60], also exhibit differences when compared to the LHCs of higher plants; in diatoms, FCP complexes are the main light-harvesting antennae. Moreover, the attribution of the different FCPs to the two photosystems and/or their supramolecular structure remains unknown. However, when comparing plant LHCs with FCPs the most obvious difference is in pigmentation.

Fx is found in much higher amounts in FCPs than carotenoids are in LHCII: the molar Chl/carotenoid ratio is almost 1:1 and 14:4, respectively [52,61]. Upon binding to the protein, Fx undergoes extreme bathochromic shifts, and since it depends strongly on the polarity of the protein environment, several populations can be distinguished, *i.e.*, Fx red, Fx green, and Fx blue (Figure 3) [62,63,64]. In diatoms, the Ddx pool is heterogeneous. Recently, three different pools of diadinoxanthin cycle pigments were proposed. Two of them are bound to special antenna proteins within PS I and FCP, respectively, and since their turnover is very low, they play no direct role in the Ddx cycle [27]. The largest pool which would be localized within an MGDG shield surrounding the FCP (Figure 3) [65,66] is convertible to Dtx during the Ddx cycle. This spatial separation has been explained in terms of the probable functional heterogeneity of these pigments. The protein-bound diadinoxanthin cycle pigments would participate in the non-photochemical quenching (NPQ) mechanism, while the lipid-associated ones would essentially play an antioxidant function, scavenging ^1^O_2_ and peroxylipids. Interestingly, Alexandre and co-workers [51] reported that the additional Ddx molecules observed when cells are grown in high light conditions adopt a more twisted conformation than the lower levels of Ddx present when the cells are grown in low light conditions. They conclude that this pool of Ddx is more tightly bound to a protein-binding site, which must differ from the one occupied by the Ddx present in low light conditions.

In the thylakoid membranes of diatoms, other xanthophylls like Vx, Ax, and Zx could also occur [24,27]. However, these carotenoids accumulate only under specific conditions, e.g., during long-term illumination with strong light. Moreover, it has been shown that Vx can be either a direct or an indirect (through the formation of Ddx) precursor of Dtx (see Section 3).

A structural homology model based on LHCII structure and spectroscopic analyses was recently published by Gundermann and Büchel [60]. It is based on the previously postulated one by Eppard and Rhiel [67] with five conserved Chl *a* binding sites (a602, a603, a610, a612, and a613; nomenclature according to Liu *et al.* [68]) and modified after Premvardhan *et al.* [62], who identified two further conserved binding sites: a614 and b609. The model accounts for some major requirements listed in [60] as follows: (i) the pigment content of FCPa and FCPb is based on 2 Chl *c* per monomer, *i.e.*, the FCPs contain 6–8 Chl *a*: 2 Chl *c*: 5–6 Fx in total; (ii) the Chl *a* molecules cannot be arranged in a way as to favor excitonic interactions; (iii) Fx does not transfer energy to Chl *c* and Fx and Chl *c* should thus be at an appropriate distance and/or orientation; (iv) Chl *c* to Chl *a* transfer is extremely fast, *i.e.*, Chl *c* has to be in close proximity to a Chl *a* molecule. For further details the reader is referred to the text [60].

## 6. Pigments Involved in Photosynthesis

**Figure 4:**
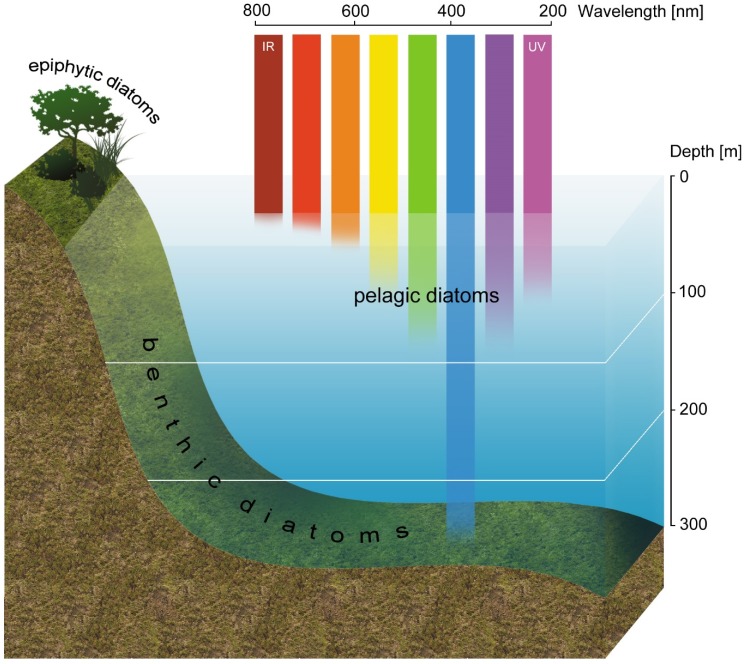
Spectra composition of light depending on the depth.

Diatoms are responsible for about 40% of marine productivity and, because the oceans cover about 70% of the Earth’s surface, they make a great contribution to global productivity [69]. However, the marine environment, especially for planktonic species, is changing continuously because of water turbulence. The cells are exposed to varied light intensities as well as light spectrums, depending on the depth (Figure 4). The cell morphology and physiology are strongly connected to the types of habitats [70,71,72], which have a great importance, especially in ecological studies based on organisms in natural environments. The most common diatom species with their typical habitats and lifestyles are described in Table 2. Consequently, diatoms are well adapted to these conditions through efficient photon accumulation and CO_2_ uptake and also through fast response to strong light to prevent photodamage. Moreover, according to Falkowski and Knoll [73], the ecological success of diatoms as one of the most important groups of planktonic species is related to their pigment profile, including Chl *a*, Chl *c*, β-car, Ddx, Dtx, and Fx, which enables them to harvest light more efficiently than do green [74,75] and red algae [74]. Chlorophylls play a light-harvesting role and are also known for their electron transfer function. This applies to Chl c, which serves exclusively as an antenna pigment. However, free monomeric Chls can easily transmute into an excited state, inducing free radicals. Therefore, the closely localized carotenoids Ddx and Dtx in diatoms are able to quench it efficiently [36].

**Table 2:** Ecological specification of the most common diatom taxa, cell morphology, colony lifestyle, habitats. The presence of species in ecological region is variable and dependent on, e.g., the season, nutrient availability, salinity, and conductivity; however, the most frequent occurrence is specified. One group is benthic species including epiphytic (attached to plants), epilithic (attached to rock surfaces), epipelic (on mud), and epipsammic (on sand) species and the second is pelagic diatoms (free living in the water column) [76,77].

Species	Morphology	Colony-Forming	Lifestyle	Habitat
*Actinella punctata*	eunotioid	yes	benthic	acidic, humic lakes, and ponds
*Actinocyclus normanii*	centric	no	planktonic	coasts, brackish waters, sediment core
*Amphora minutissima*	asymmetrical biraphid	no	benthic	marine habitats, often epiphytic
*Bacillaria paradoxa*	nitzschioid	yes	benthic	marine, brackish, and freshwaters
*Campylodiscus hibernicus*	surirelloid	no	benthic	epipelon in fresh, brackish, marine waters
*Cocconeis pediculus*	monoraphid	no	benthic	planktonic, epiphytic, epilithic habitats
*Cyclotella distinguenda*	centric	no	planktonic	preferentially alkaline waters
*Cymbella amplificata*	asymmetrical biraphid	yes	benthic	oligotrophic waters
*Diatoma vulgaris*	araphid	yes	benthic	fresh and brackish water
*Discostella stelligera*	centric	no	planktonic	primarily in lakes and large rivers
*Distrionella incognita*	araphid	yes	benthic	alkaline lakes and streams
*Epithemia turgida*	epithemioid	no	benthic	epiphyte on coarse filamentous algae
*Eucocconeis alpestris*	monoraphid	no	benthic	the littoral zone of oligotrophic lakes
*Eunotia exigua*	eunotioid	N/A	benthic	moist soils, wet walls, streams, waterfalls
*Fragilaria crotonensis*	araphid	yes	planktonic	mesotrophic lakes, water column
*Gyrosigma acuminatum*	symmetrical biraphid	no	benthic	primarily an epipelic species
*Navicula reinhardtii*	symmetrical biraphid	no	benthic	fresh water, slightly brackish
*Nitzschia regula*	nitzschioid	no	benthic	cold-water, ponds, and streams
*Phaeodactylum tricornutum*	fusiform, triradiate, oval	no	planktonic	marine coastal waters
*Pinnularia rabenhorstii*	symmetrical biraphid	no	benthic	cold oligotrophic waters in the mountains
*Pleurosira laevis*	centric	yes	benthic	naturally saline or polluted waters
*Thalassiosira weissflogii*	centric	N/A	planktonic	primarily in marine waters

Studies on acclimation to different light conditions, not only in terms of the quantity but also the quality of light, have been conducted. It was observed that during daily insolation the Chl content increased and it was accompanied by a decrease in the Fx content [78]. Both pigments are present in FCP complexes and have different absorption maxima, which results in efficient photon accumulation. Although the ratio between light harvesting and photoprotective pigments is balanced and, therefore, photosynthetic efficiency is lowered under high irradiance, because the amount of alternative electrons is kept to a minimum under fluctuating light, diatoms can convert energy into biomass efficiently [78]. Additionally, there is valuable data about the adaptation of diatom cells to their environment, achieved through experiments with selected light spectra. Blue light has a significant impact on diatoms, resulting in the production of more cells with greater photosynthetic activity in comparison to white light [79]. Moreover, planktonic diatoms which float in the water column are exposed to changes in the ratio between blue and red light. Recently, Jungandreas and co-workers [45] found that a shift from blue to red light results in a slightly diminished growth rate within 48 h, whereas a shift from red to blue light completely abolished growth within 24 h. Furthermore, metabolic reorganization based on the carbon allocation in diatom cells is the first response in acclimation to new light conditions [45]. Beyond light stress, another group of factors such as nutrient availability have a substantial impact on photosynthetic efficiency. Silicon is a special inorganic compound in diatoms, occurring abundantly in their cell wall. The energy for silicification is more linked to respiration than to photosynthesis and, therefore, it has no effect on oxygen production but leads to decreased utilization of carbohydrates during biomineralization [2]. It has been reported that silicon limitation results in increased Chl *a* content [80,81], but its addition had no significant effect on Chl accumulation [82]. Nevertheless, silicate importance for photosynthetic efficiency is similar to other nutrients [83], including iron, which plays an important role in marine primary productivity. A decreased Chl content, lowered growth rate, up-regulated expression of genes engaged in early steps of carotenoid biosynthesis, and enhanced NPQ were observed under iron deficiency [49], which altogether resulted in a down-regulation of photosynthesis, nitrate assimilation, and mitochondrial electron transport.

Although photosynthetic pigments are related to processes depending on light, dark phase reactions are equally important. Diatoms might perform both C_3_ as well as C_4_ photosynthesis and, indeed, both types of initial products were found in one species, and it was reported that the CO_2_ concentration has no effect on the transcripts of genes engaged in this process [84]. As in other photosynthetic organisms, the enzyme responsible for carbon fixation is RubisCO, which also has the ability to fix oxygen. Additionally, diatoms’ cytoplasmic membrane is highly permeable and a lot of CO_2_ is lost by diffusion. However, diatoms are well adapted to low CO_2_ concentrations through an efficient CO_2_ concentration mechanism based on the active transport of carbon from the cytoplasm into the chloroplast [85]. As described above, despite environmental fluctuations, diatoms are well-adapted marine organisms with great importance to global productivity.

## 7. Pigments Involved in Photoprotection

Light conditions change significantly depending on the daily cycle, season, habitat, and environment. These conditions determine the life of photosynthetic organisms that have to react to differing situations, accumulate photons efficiently in low light, and dissipate excesses of energy in high light. Excess light might lead to photoinhibition, inactivation of photosystems, and induce the formation of reactive oxygen species (ROS), resulting in photodamage and wide-ranging changes in the cells caused by oxidative stress. To avoid this, phototrophs have developed photoprotective mechanisms such as NPQ and the xanthophyll cycle.

The NPQ relies on the dissipation of excessive excitation energy as heat and might be detected as a quenching of Chl *a* fluorescence. Various mechanisms have been postulated to describe this process; however, all of them require structural changes in photosynthetic antenna that allow a transition from light harvesting to an energy dissipation state [86]. The role of the proton gradient between thylakoid lumen and chloroplast stroma, as well as the presence of xanthophylls and the LHCII structure, have all been thoroughly investigated in higher plants (for a review see [86]), but there has also been significant progress in this field when it comes to diatoms. Studies carried out so far indicate the importance of Dtx in NPQ that is observed only in the presence of this pigment. Contrary to plants and green algae, the proton gradient across the thylakoid membrane in diatoms is not sufficient to induce NPQ [87]. Furthermore, the decrease in the proton gradient does not result in NPQ relaxation, which is correlated with Dtx epoxidation [88].

**Figure 5:**
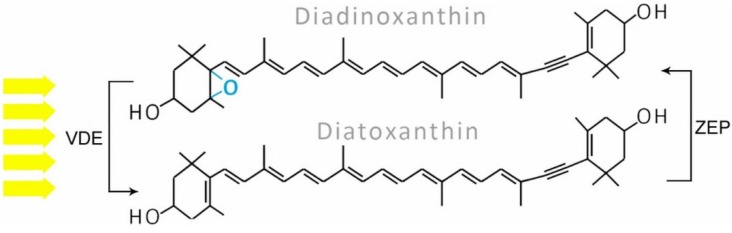
The diadinoxanthin cycle: in high light, diadinoxanthin with one epoxy group is converted to epoxy-free diatoxanthin by violaxanthin de-epoxidase (VDE); the reverse reaction is observed in low light and dark and is catalyzed by zeaxanthin epoxidase (ZEP).

**Figure 6:**
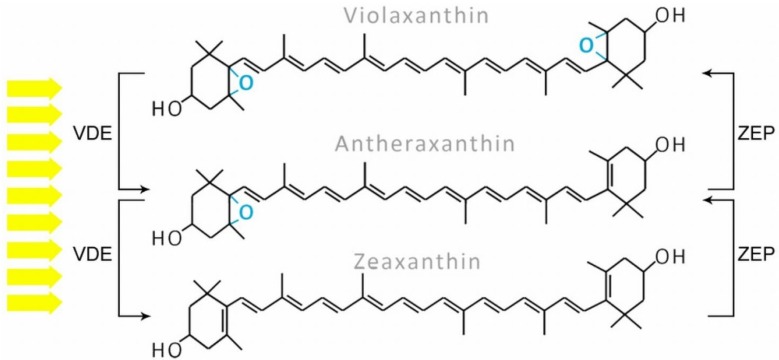
The violaxanthin cycle: under high light, violaxanthin (which is normally a precursor of fucoxanthin) is converted to zeaxanthin via the intermediate antheraxanthin and this reaction is catalyzed by violaxanthin de-epoxidase (VDE), whereas in low light and dark, two single steps of oxygenation catalyzed by zeaxanthin epoxidase (ZEP) lead to violaxanthin formation.

Six types of xanthophyll cycles have been described in photosynthetic organisms and two of them occur in diatoms [89]. Typical of this group is the diadinoxanthin cycle (DD cycle, see Figure 5), which exists in algae such as diatoms, phaeophytes, dinophytes, and haptophytes. The two pigments engaged in this process, Ddx and Dtx, are normally present in their cells, although their amount depends on the light intensity (see Section 8). In high light, Ddx, which is a monoepoxide xanthophyll, is converted to epoxy-free Dtx by Ddx de-epoxidase (DDE, also called VDEs, three isoforms were even identified); in low light or dark conditions there is the reverse reaction, leading to Ddx formation which is catalyzed by Dtx epoxidase (DEP, also called ZEPs, including three isoforms). Overlong light stress leads to the formation of Zx, one of three xanthophylls involved in the violaxanthin cycle (VAZ cycle), as shown in Figure 6, which occurs mostly in higher plants, mosses, and lichens, but in several conditions also observed in diatoms [24]. It relies on a cyclic conversion of di-epoxy Vx and epoxy-free Zx via an intermediate product, mono-epoxy Ax. De-epoxidation of Vx is catalyzed by VDEs and epoxidation by ZEPs. Nevertheless, it is still not known which of the three isoforms of both enzymes identified in *P. tricornutum* are engaged in the DD or VAZ cycle.

Studies on ZEPs and VDEs in this species have shown that their transcript levels vary (the lowest for ZEP3 and VDL1) and are stimulated by white and blue light [31], but there is no data on their correlation with the type of xanthophyll cycle. Although there are similarities of these enzymes in diatoms and higher plants based on structure, substrate, and cosubstrate requirements, the properties and the mechanism of light-dependent activation/deactivation are not yet understood. Epoxidation in diatoms under low light is faster than those in higher plants and green algae [88]. Although it is known that this reaction in higher plants occurs in both light and dark conditions, in diatoms, the presence of a proton gradient between thylakoid lumen and chloroplast stroma in high light almost completely inhibits epoxidation [88,90]. In *P. tricornutum*, the rate of Ddx de-epoxidation is higher than that of Vx, whereas Dtx and Zx epoxidation rates are similar [24]. Moreover, VDEs in diatoms are active at higher pH values than those in plants and need a lower ascorbate concentration as a cosubstrate [91,92]. Although the photoprotective role of both xanthophyll cycles as well as the ability of Dtx and Zx to quench excited Chl and free radicals are known, the reason why they exist together in diatom cells and the activation mechanism of each remain unclear. Indeed, Zx might be formed from both Vx and β-car and de-epoxidation, and *de novo* carotenoid synthesis could play a substantial role in its accumulation under high light [24]. It is interesting that in *Chlamydomonas reinhardtii* with no functional xanthophyll cycle, the accumulation of Zx was observed, which indicates the crucial role of β-car as a substrate [93] and diminishes the importance of the VAZ cycle. Moreover, the possibility that Zx may not be an obligate precursor of Vx was put forward when Cx and CxE were detected in *P. tricornutum*, although conversions between β-car, Cx, CxE, Vx, Ax, and Zx require detailed studies [27]. A comparative analysis of the photoprotective properties of Zx and Dtx is needed. The quenching efficiency of Dtx has been tested and it was shown that this pigment is a better quencher in low light because additional molecules of Dtx and Ddx bind to FCP, and while not participating in NPQ, they are precursors of Fx [94]. This may suggest that Zx is necessary to avoid photodamage in high light and could explain its accumulation.

## 8. Methods of Pigment Analysis and Identification

Photosynthetic pigments are used as indicators of the relative abundance of major microalgal taxonomic groups and offer a means for assessing changes or differences in the relative abundance of phototropic functional groups in mixed assemblages [95]. This approach provides measurements of the quantitative changes in the relative abundance of major microalgal groups under similar environmental conditions and habitats. As reported by Schagerl and Kunzl [96], within the year 2005, in nine international journals, 15 different concentrations and six solvents were used, whereas pigment quantification was done by measuring pigment extracts by means of fluorometry (35%) and spectrophotometry (35%), followed by HPLC (25%). Given the fact that Chl *a* is extensively used as an indirect measure of overall algal biomass, and other pigments like Chl *c* or carotenoids have been successfully applied as markers for specific taxonomic groups within algal communities [96] and the references therein, the varieties of methods make comparisons very difficult. Although the determination of chlorophylls is routinely conducted by spectrophotometric [97] or spectrofluorometric [98] methods, the results may be erroneous due to the fact that the absorption and emission bands of Chl *a* overlap with those of other Chls and the degradation products of chlorophylls are neither detected nor determined along with their parent chlorophylls. Moreover, the determination of individual carotenoids is difficult to achieve by these methods. Thus, pigment analysis by HPLC has become the favorite tool for marine researchers.

### 8.1. Extraction of Pigments

The best extraction technique is still a matter for debate. The efficiency of pigment extraction may vary depending on the properties of the solvent, the duration of the extraction, cell concentration, species of algae, and whether mechanical disruption is used. Physical methods of sample disruption such as grinding, bath sonication, high power sonication, or soaking are in use and the most suitable solvents currently applied include acetone, methanol, and non-volatile *N*,*N*-dimethylformamide (DMF) (see [96] and the references therein). The need for the complete extraction of all pigments present in the sample, compatibility with chromatographic technique, and stability of the pigments are to be considered before the choice is made.

The application of various extraction solvents has been thoroughly discussed by Wright and co-workers [99], of which both acetone and methanol are widely applied in the extraction of algal pigments. Although the highest extraction efficiency is generally achieved by using methanol as an extraction solvent in combination with mechanical disruption, the stability of pigments in methanol is low. It has often been claimed to promote allomerization of Chl *a* [100,101] and, accordingly, Mantoura and Llewellyn [102] found that methanol led to the formation of Chl *a*-derivative products. On the other hand, there is evidence that certain chlorophylls and carotenoids are more thoroughly extracted with methanol [103] or dimethyl sulfoxide [104]. Altogether, acetone seems to be the best choice when there is no information on the specific composition of the sample. While acetone and methanol have the same polarity index, acetone has greater eluotrophic strength than methanol for carbon-rich substrates [105]. Moreover, it has been proved to cause fewer artifacts than methanol and DMF as the derivatives of Chl *a* (mainly Chlide *a*, Chl *a* allomer and epimer) were found at only up to 5% of measured Chl *a* [96]. The extraction efficiency of acetone increases considerably when, prior to extraction, freeze-drying is applied, which breaks up the protein matrix of membranes, creating accessibility for the extraction solvent even with more recalcitrant algae [100,106]. Additionally, freeze-drying inhibits the activity of chlorophyllase, the enzyme found in many marine taxa [101,107], which catalyzes the hydrolysis of the bond linking the phytol chain to the propionic acid residue of Chls *a* and *b*, thereby converting Chl to its corresponding chlorophyllide [108,109]. Since chlorophyllase is mainly active in an aqueous environment [108], it appears that dehydration by freeze-drying creates conditions that inhibit enzyme activity. To conclude, it is worth remembering that algal pigments are extracted differentially by various solvents, and there is no single combination of solvent and extraction time that is best for all species [103].

### 8.2. HPLC Analysis of Pigments

HPLC was applied to phytoplankton studies in the early 1980s [110]. All current methods use reverse phase separation where compounds are resolved primarily on the basis of their polarity. Up until now, different columns have been tested, including octadecylsilica (ODS) C18 and octylsilica (OS) C8, as well as C30 stationary phase, with different diameters, lengths, and particle sizes, which provide different degrees of effectiveness in the resolution of key pigments. Pigment separation and resolution can be also tuned by the column temperature, mobile phase composition, and the gradient program. For the resolution of acidic chlorophylls, buffering, ion-pairing, or an ion suppression reagent is required, with ammonium acetate as the one of choices.

**Figure 7:**
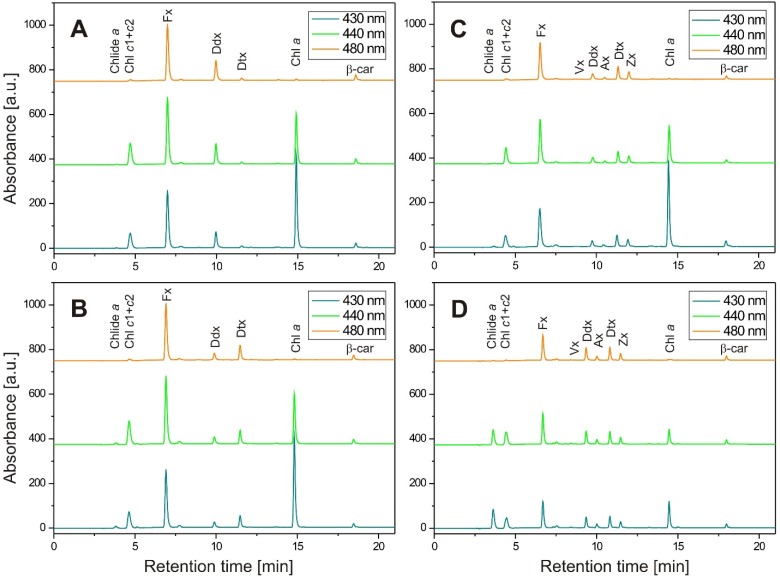
Examples of HPLC chromatograms of absorbance recorded at 430, 440, and 480 nm obtained with the method by Kraay and co-workers [117] for pigment extracts from the diatom *P. tricornutum*: (**A**) 1 h dark incubated cells growing in LL; (**B**) 1 h HL illuminated cells growing in LL; (**C**) 1 h HL illuminated cells growing in ML; (**D**) 48 h HL illuminated cells growing in LL. LL: white light with the intensity of 100 μmol m^−2^·s^−1^ in a 16 h light/8 h dark photoperiod; ML: white light with the intensity of 700 μmol m^−2^·s^−1^ in a 6 h light/18 h dark photoperiod; HL: white light with the intensity of 1250 μmol m^−2^·s^−1^.

**Figure 8:**
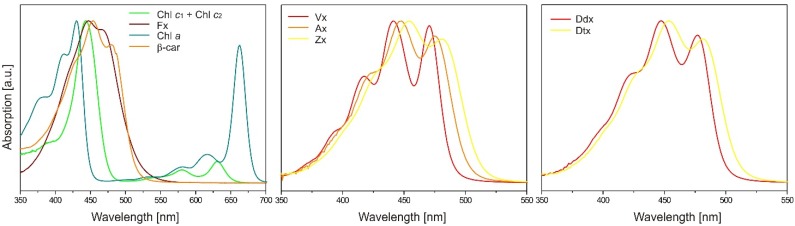
Absorption spectra of photosynthetic pigments recorded during HPLC-DAD analysis performed on extracts from the diatom *P. tricornutum* with the method by Kraay and co-workers [118]. The spectra were normalized at λ_max_.

A comprehensive monograph where both modern analytical methods and their applications to biological oceanography were reviewed is that of Jeffery and co-workers from 1997 [111] and 1999 [112]. Nineteen HPLC methods published between 1983 and 1998 have been reported. In short, the work has been started by Mantoura and Llewellyn [102], who developed a reverse-phase HPLC technique for the rapid separation and quantification of 17 carotenoids and 14 chlorophylls together with their breakdown products from acetone extracts of algal culture and natural waters. They also suggested that the presence of an ion-pairing reagent during the chromatographic separation was essential to obtain good resolution for chloropigments (chlorophylls, chlorophyllides, and phaeophorbides). A modified technique was described by Wright and Shearer [113] in which photosynthetic pigments from phytoplankton were separated by HPLC using a lineal elution gradient from 90% acetonitrile to ethyl acetate. Although a better resolution was obtained by the latter method for carotenoid pigments, it does not resolve the polar chlorophylls *c*_1_, *c*_2_, *c*_3_, and Mg-2,4 divinyl phaeoporphyrin *a*_5_ monomethyl ester (Mg2,4D), which coelute as a single peak, or adequately resolve the chlorophyllides *a* and *b*. A more comprehensive method was published in 1991 [114], and has been used thereafter for routine work. Further improvement has been achieved by replacing ammonium acetate with either a pyridine or tetrabutylammonium (TBAA) modifier. In particular, Zapata and co-workers [115], using a gradient from aqueous methanol/acetonitrile to methanol/acetonitrile/acetone with a pyridine modifier (as the acetate, pH 5.0), succeeded in resolving, on a C8 column, seven polar Chl *c* derivatives, the Chl *a*/DV Chl *a* pair, and, partially, Chl *b*/DV Chl *b*. The method of Van Heukelem and Thomas [116] is based on an aqueous methanol to methanol gradient at 60 °C with a TBAA modifier (pH 6.5). The effectiveness of two HPLC methods (a monomeric C18 column with a high ion strength mobile phase composed of methanol, acetonitryl, and ethyl acetate by Kraay and co-workers [117] (Figure 7 and Figure 8) and a monomeric C8 column with a pyridine-containing mobile phase [116]) in the separation, identification, and quantification of pigments were compared by Mendes and co-workers [118], using phytoplankton, microphytobenthos, and algal cultures. Although the C8 method allows the separation of chlorophylls *c*_1_, *c*_2_, and Mg-2, 4-divinyl phaeoporphyrin *a*_5_ monomethyl ester, as well as the pair Chl *a*/DV Chl *a*, the C18 method had a shorter elution program and a lower solvent flow rate, making it cheaper and faster than the C8 method. The excessive separations feasible with C30 columns have proven useful for unique applications such as pigment isolation [116], although the long analysis times are a deterrent if routine separations of complex mixtures are required.

For the detection and quantitative analysis of pheophytin *a*, pheophorbide *a*, and their derivatives, the wavelengths of 420–430 nm are useful, while the region of 450–480 nm detects all carotenoids and Chl *c* without interference from Chl *a* derivatives. Peaks can be quantified either using internal or external standards. The first approach ensures higher accuracy as it accounts for any changes resulting from evaporation or dilution as well as providing a check on the injection status. Commercially available internal standards in common use are ethyl 8′-apo-β-carotenoate, 8′-apo-β-carotenoal, vitamin E, and canthaxanthin, the first of which attracts special interest due to its stability and non-occurrence in natural systems. Table 3 shows the elution order of photosynthetic pigments from the diatom *P. tricornutum*, analyzed with the method by Kraay and co-workers [117], and their visible absorption characteristics. The absorbance spectra of more algal pigments and their elution order are available in the literature [53,102,114,118,119].

Pigment identification can be confirmed by performing LC/MS and comparing the resulting mass spectra with published fragment ion abundance and/or mass spectral libraries of pigment standards.

**Table 3:** Elution order of photosynthetic pigments from the diatom *P. tricornutum* and their visible absorption characteristics.

Pigment	Literature Data				HPLC Data *	
	Solvent	λ_max_ (nm)	E (L g^−1^·cm^−1^)	Reference	λ_max_ (nm)	Retention Time (min)
Chlorophyll *c*_1_	acetone (90%)	443	318	[120]		
Chlorophyll *c*_2_	acetone (90%)	444	374	[120]		
Chlorophyll *c*_1_ + *c*_2_	N/A	N/A	N/A	N/A	443	4.7
Fucoxanthin	petrol ether	449	165	[121]	447	7.0
	ethanol	450	114	[122]		
	acetone	443	165	[123]		
Violaxanthin	N/A	443	N/A	[114]	441	8.4
Diadinoxanthin	acetone	448	224	[124]	447	9.9
	methanol	445	225	[124]		
	hexane	446	211	[124]		
Anteraxanthin	ethanol	446	235	[125]	447	10.8
Diatoxanthin	acetone	454	N/A	[114]	454	11.3
Zeaxanthin	ethanol	452	254	[126]	453	11.9
Chlorophyll *a*	acetone	662	88.15	[127]	429	14.9
β-carotene	etanol	453	262	[128]	454	18.7
	hexane	453	259.2	[128]		
	acetone	454	250	[129]		

*: data obtained with the method by Kraay andco-workers [117] for pigment extracts from the diatom *P. tricornutum*.

## 9. Significance of Diatom Pigments

Marine algae are rich sources of structurally diverse bioactive compounds with various biological activities. Among them, natural pigments have received particular attention as they exhibit beneficial activities such as antioxidant, anticancer, anti-inflammatory, anti-obesity, anti-angiogenic, and neuroprotective effects important in the fields of food, cosmetics, and pharmacology [130]. Although chemically synthesized pigments are widely available, natural products are more attractive to consumers as they exhibit higher bioavailability and bioactivity. Since bioreactors allow successful cultivation of different species of marine microorganisms, including diatoms, the latter are considered a good source of natural pigments. A relatively fast growth rate along with with low costs is by far the best advantage; however, it has to be taken into account that pigments for food or pharmaceutical applications need to be of a consistently high quality that is safe, traceable, measurable, and readily extractable, which makes the technology more demanding. Nevertheless, the great health benefits and nutraceutical properties represented by diatom pigments make them interesting for pharmaceutical companies.

Carotenoids, especially β-car as the most efficient natural quencher, are involved in the scavenging of ROS, singlet molecular oxygen, and peroxyl radicals, so they may protect against lipid peroxidation [131]. β-car is also an effective stimulator of gap junctional communication, which is associated with cell growth, differentiation, and apoptosis and, together with α-tocopherol, inhibits the lipid peroxidation better than on its own [132]. The anticancer activity of β-car was confirmed, e.g., for lung cancer, although the risk of cancer increased when high doses of a dietary supplement rich in this carotenoid were consumed by smokers [133]. Lutein and Zx are responsible for the coloration of the macula lutea, which is a part of the retina, the area of maximal visual acuity; thus, both pigments are extremely important in ophthalmology and are also used in protection against age-related macular degeneration [134]. This photoprotection arises from their function as filters for damaging blue light and as quenchers of excited oxygen. Strong antiproliferative activity of Vx on breast cancer cells was demonstrated, which makes this pigment a potent compound of drugs for the induction of apoptosis [135]. Although diatoms contain a few photosynthetic pigments including chlorophylls and carotenoids, Fx has the greatest significance in these organisms. This xanthophyll is present in large amounts in diatoms and brown algae, whereas Zx, Ax, Vx, and β-car are sourced mainly from plants where they are present abundantly. Nevertheless, two other xanthophylls, Dtx and Ddx, are also specific to diatoms and might be obtained from them. Unfortunately, the biological activity of their extracts, health benefits, and importance in the diet are still unknown and should be a major motivation for future research.

Fx has great commercial importance because of its varied and numerous bioactivities, such as antioxidant, anticancer, anti-inflammatory, anti-obesity, anti-angiogenic, neuroprotective, photoprotective, and prevention of osteoporosis [130]. The extracts of a few seaweed species such as *Hyaleucerea fusiformis*, *Cladosiphon okamuranus*, *Undaria pinnatifida*, and *Sargassum fulvellum* showed strong DPPH radical scavenging activity [136]. Fx may inhibit intracellular ROS formation, DNA damage, and apoptosis induced by H_2_O_2_ and its antioxidant activity is comparable to that of α-tocopherol [136]. The anti-inflammatory activity of Fx is associated with its ability to reduce the nitric oxide level, prostaglandin E2, tumor necrosis factor-α, interleukin-1β, interleukin-6, and histamine [137,138]. Strong antiproliferative activity is exhibited by Fx which may induce the apoptosis of human leukemia cell line HL-60, and reduce the viability of PC-3 human prostate cancer cells and human colon cancer cell lines Caco-2, HT-29, and DLD-1 [139,140] (for a review see [136]). Moreover, Fx is a promising compound that might be used in obesity prevention through the upregulation of UCP1 which promotes energy expenditure by thermogenesis [141]. It was reported that Fx mixed with pomegranate seed oil reduced body weight, body fat, and liver fat content [142].

## 10. Methods of Purification of Diatom Pigments

From among photosynthetic pigments occurring in diatoms, neither chlorophylls nor β-car are of interest to be extracted and purified on a preparative scale since they are more abundant in plants. On the contrary, the broad application potential of Fx has prompted researchers to explore the commercial production of this photosynthetic pigment from algae. To find an efficient source of Fx, brown macroalgae, such as *Laminaria japonica*, *Eisenia bicyclis*, and *Undaria pinnatifida* [143,144], were studied; they are traditional foods in Southeast Asia and some European countries. However, the Fx concentration from these macroalgae ranged from 0.02 to 0.58 mg·g^−1^ in fresh samples and 0.01 to 1.01 mg·g^−1^ in dried samples [26], which makes the production of Fx from brown macroalgae not commercially viable. Since the reported Fx concentration in microalgae is one to three orders of magnitude greater than that found in macroalgae, ranging from 2.24 to 18.23 mg·g^−1^ [26], diatoms are considered a promising source of Fx for various commercial applications. In particular, *P. tricornutum* was found to contain Fx as a main carotenoid. Studies by the Rebolloso-Fuentes research group [145] showed that 60% of the total carotenoids in the acetone extract was Fx, which gives Fx a concentration of 1.81 mg/g dw. Xia and co-workers [8] investigated the marine diatom *Odontella aurita* and determined that it can accumulate high concentrations of Fx (>20 mg·g^−1^ of dry weight). The Fx concentration of 3.33–21.67 mg·g^−1^ and the volumetric concentration of 18.15–79.56 mg·L^−1^ obtained in this study have set the records for algae-based Fx production. A dataset containing the Fx concentrations in samples of different macroalgae and microalgae is available in [8,26].

There are numerous methods used to recover and purify Fx from brown algae, including: (i) centrifugal partition chromatography; (ii) pressurized liquid extraction; (iii) enzymatic pre-treatment followed by co-solvent extraction; (iv) supercritical carbon dioxide with ethanol as co-solvent; and (v) traditional solvent extraction followed by chromatographic methods, as reviewed by Gomez-Loredo in [146]. However, the costs associated with new technologies and equipment are major drawbacks to their implementation. Thus, there is a need to develop low cost, simple, and scalable strategies for the recovery of Fx as one of the high value compounds occurring naturally in abundance in diatoms. The results show that Fx extraction efficiency, as with other phytoplankton pigments, is highly dependent on the extraction conditions—the solvent type, in particular (see Section 8). Kim and co-workers [26] have assessed a number of extraction procedures to investigate the effect of solvent type, extraction time, temperature, and extraction method (maceration, ultrasound-assisted extraction, Soxhlet extraction, and pressurized liquid extraction) on Fx extraction efficiency. Among the investigated solvents, ethanol provided the best Fx extraction yield (15.71 mg g^−1^ freeze-dried sample weight), whereas its content in the extracts produced by the different methods was quite constant (15.42–16.51 mg·g^−1^ freeze-dried sample weight). The pigment extracts obtained from freeze-dried powder of *P. tricornutum* extracted with 100% ethanol at room temperature for 3 h were then subjected to silica-gel adsorption chromatography with an eluent of n-hexane:acetone (7:3, v/v). These results are in accordance with findings reported by Xia and co-workers [8], who additionally pointed to the fact that selecting a proper ratio of solvent to dry algal biomass (v/w) is important, as it may affect the quantity and quality of Fx. In particular, they showed that an ethanol/dry biomass ratio of 20:1 is sufficient for the effective extraction of Fx from this microalga. In this work, the crude pigment extracts were subjected to open silica gel column chromatography with n-hexane:acetone (6:4, v/v) as the eluting solvent system. An orange-red colored Fx-rich fraction containing Fx, *cis*-Fx, Ddx, and Dtx was separated by the column. Further purification of Fx, whose purity was estimated as 86.7% in the mixture, was carried out by a prep-HPLC yielding a final Fx purity >97%.

Alternatively, the two-phase solvent system of n-hexane:ethanol:water with a volume ratio of 10:9:1 was determined to be the best system for the separation of Fx and lipids from extracts of *Isochrysis galbana*. Under these conditions, Fx was fractionated in the hydroalcohol phase apart from the hexane phase containing lipids [147]. Recently, Gómez-Loredo and co-workers [146] proposed a different potential strategy for the recovery and partial purification of Fx. In their study, the partition behavior of Fx in aqueous two-phase systems (ATPS) composed of ethanol and potassium phosphate was evaluated. The optimal extraction conditions were found to be un-acidified methanol (2.5% w/v for *P. tricornutum*) or ethanol (5% w/v for *P. tricornutum*) as a solvent with orbital agitation at 250 rpm for 1 h at 20 °C. The best results were observed in systems with a tie-line length, TLL 50% w/w, and a volume ratio of 1 for *P. tricornutum* methanolic extracts (95.36% recovery and 66.01% purity).

To the best of our knowledge, there is no method published so far to obtain Ddx and Dtx on a preparative scale, except for a semi-preparative HPLC approach. Thus, the method based on column chromatography recently elaborated in our laboratory (see Acknowledgments) seems to be promising for the potential application of these xanthophylls naturally found in diatoms.

## 11. Conclusions

Apart from chlorophylls *a* and *c*, and fucoxanthin, which play a light-harvesting function, a group of photosynthetic pigments in diatoms comprises those engaged in photoprotection, including β-carotene, diatoxanthin, diadinoxanthin, violaxanthin, antheraxanthin and zeaxanthin. Physical and chemical properties of diatom pigments have been extensively studied, which contributed to a more complete understanding of the function they play in cells as well as enabled explanation of their health promoting activity and beneficial effects. There are, however, still further studies required to gain the knowledge on Chls *c* to a level comparable with the well-studied Chls *a* and *b*. Moreover, it is of great importance to explore biosynthetic pathways of Chls *c* and xanthophylls by identifying the enzymes involved and the specificity of their reactions. There is a growing body of studies on diatom pigments, which refers to their photosynthetic and photoprotective functions and explains the mechanisms of these processes. Nevertheless, there are still many questions to be answered in the field of xanthophyll cycle and non-photochemical quenching research. Photoacclimation and pigment level variation are also commonly studied, which is related to the fact that marine environment is highly changeable. Furthermore, analysis of diatom pigments is widely used in global oceanography to assess phytoplankton biomass, productivity, community structure and ecological processes. Natural pigments, including those derived from algae have received particular attention as they exhibit beneficial activities important in terms of their commercial and industrial applications. In this respects, diatoms seem to be a promising source of unique bioactive compounds, with fucoxanthin, diadinoxanthin and diatoxanthis as representatives of photosynthetic pigments.

## Abbreviations

Ax: antheraxanthin; ATPS: aqueous two-phase systems; BCH: β-carotene hydroxylase; BHT: butylated hydroxytoluene; Chl *a*: chlorophyll a; Chl *b*: chlorophyll b; Chl *c*: chlorophyll c; Cx: β-cryptoxanthin; CxE: β-cryptoxanthin epoxide; DD cycle: diadinoxanthin cycle; Ddx: diadinoxanthin; DMAPP: dimethylallyl diphosphate; DMF: *N*,*N*-dimethylformamide; DPOR: light-independent PChlide oxidoreductase; Dtx: diatoxanthin; FCP: fucoxanthin-chlorophyll protein; Fx: fucoxanthin; GGPP: geranylgeranyl pyrophosphate; HPLC: high performance liquid chromatography; IPP: isopentenyl pyrophosphate; ICT: intramolecular charge transfer; LC/MS: liquid chromatography/mass spectrometry; LCYB: lycopene β-cyclase; LHC: light harvesting complex; LPOR: light-dependent PChlide oxidoreductase; MEP: methylerythritol phosphate; MEV: mevalonate; MGDG: monogalactosyldiacylglycerol; NPQ: non-photochemical quenching; Nx: neoxanthin; NXS: neoxanthin synthase; ODS: octadecylsilica; OS: octylsilica; PChlide: protochlorophillide; PDS: phytoene desaturase; PS I: photosystem I; PSY: phytoene synthase; ROS: reactive oxygen species; RubisCO: ribulose bisphosphate carboxylase-oxygenase; TBAA: tetrabutylammonium; TLL: tie-line length; VAZ cycle: violaxanthin cycle; VDEs (VDE, VDL1, VDL2): violaxanthin de-epoxidases; Vx: violaxanthin; ZDS: ζ-carotene desaturase; ZEPs (ZEP1, ZEP2, ZEP3): zeaxanthin epoxidases; Zx: zeaxanthin; β-car: β-carotene.
